# Evaluating the impact of a low-cost food storage intervention on complementary food contamination and diarrheal disease in low-income urban households: A randomized controlled trial in Dhaka, Bangladesh

**DOI:** 10.1371/journal.pgph.0005883

**Published:** 2026-06-11

**Authors:** Musarrat Jabeen Rahman, Syeda Nurunnahar, Nazrin Akhter, Mahbubur Rahman, Mohammed Badrul Amin, Rehnuma Haque, Peter J. Winch, Laura H. Kwong

**Affiliations:** 1 Social and Behavioral Interventions Program, Department of International Health, Johns Hopkins Bloomberg School of Public Health, Baltimore, Maryland, United States of America; 2 Environmental Health and WASH, Health Systems and Population Studies Division, International Centre for Diarrhoeal Disease Research, Bangladesh (icddr, b), Dhaka, Bangladesh; 3 Global Health and Migration Unit, Department of Women’s and Children’s Health, Uppsala University, Uppsala, Sweden; 4 Food Safety and One Health Laboratory, Nutrition Research Division, International Centre for Diarrhoeal Disease Research, Bangladesh (icddr, b), Dhaka, Bangladesh; 5 Environmental Health Sciences, Kirk R. Smith Scholar, Global Environmental Health, University of California, Berkeley, California, United States of America; ICDDR B: International Centre for Diarrhoeal Disease Research Bangladesh, BANGLADESH

## Abstract

Diarrheal diseases remain a leading cause of under-five mortality globally, yet large-scale water, sanitation, and hygiene (WASH) interventions have shown limited effects on childhood diarrhea and growth, partly because foodborne transmission has been overlooked. In low-income urban settings, complementary foods for young children are often stored for several hours under unsafe conditions, increasing the risk of fecal contamination. This randomized controlled trial evaluated whether a low-cost wire-mesh food cabinet (“meatsafe”), paired with one-time behavior-change communication (BCC), could reduce microbial contamination and diarrhea among children aged 6–24 months in the Korail informal settlement of Dhaka, Bangladesh. A total of 290 households were randomly assigned 1:1 to receive a meatsafe plus food-hygiene handouts or no intervention. Over five biweekly post-intervention visits, we collected stored complementary food samples (≥4 h) for *Escherichia coli* testing and recorded 7-day caregiver-reported diarrhea. The primary outcome was the prevalence of high-level contamination (≥100 CFU *E. coli*/g). Prevalence ratios (PRs) were estimated using population-averaged modified Poisson regression adjusted for storage conditions. Across 1,545 food samples, contamination declined in both arms—from 49.6% to 25.2% in controls and 39.0% to 18.6% in the intervention arm—but the intervention had no effect on high-level contamination (adjusted PR = 0.83; 95% CI: 0.55–1.26) or caregiver-reported diarrhea (PR = 0.56; 95% CI: 0.24–1.31). Storing food for more than eight hours remained strongly associated with contamination (aPR = 1.49; 95% CI: 1.20–1.86). Although meatsafe adoption was high (88% at first visit, 99% at last), recall of general hygiene messages was limited. Providing a meatsafe with minimal BCC did not reduce food contamination or child diarrhea, despite excellent uptake. The findings underscore the limits of simple, passive technologies in resource-constrained settings and suggest that future strategies should integrate affordable storage innovations with more intensive and sustained hygiene promotion.

**Trial registration:**

ClinicalTrials.gov, NCT07332078. Registered 12 January 2026 (retrospectively registered); https://clinicaltrials.gov/study/NCT07332078.

## Introduction

Diarrheal diseases caused approximately 1.17 million deaths in 2021, including 340,429 children under five years of age. Infants aged 6–11 months in low- and middle-income countries (LMICs) endure an average of 4.5 episodes of diarrhea per year, 12% of which last a week or more and impair nutrient absorption [1–3]. Yet large-scale WASH trials in Bangladesh, Kenya, Zimbabwe, and Mozambique produced little or no impact on child growth or diarrhea [4–8]. One explanation is that the components of the intervention packages focused almost exclusively on water treatment, human feces management, and handwashing, leaving other fecal–oral pathways largely unaddressed [9].

While systematic reviews have highlighted widespread fecal contamination of complementary foods in low-income settings [10] and food is widely recognized as a central component of fecal-oral transmission [11], food hygiene has received limited attention in recent WASH interventions [4–7]. In urban Bangladesh, the odds of diarrhea was 6.16 times higher among children from households where food was stored uncovered compared covered [12]. In rural Bangladesh, stored complementary food that was uncovered at the time of sampling had twice the prevalence of contamination with ≥100 MPN/dry g compared to stored food that was covered [13]. Furthermore, consumption of microbiologically contaminated complementary food was associated with higher rates of diarrhea and malnutrition among Bangladeshi children aged 6–24 months [14] and prospective analysis in rural Bangladesh showed that detection of E. coli in food was linked to more than a two-fold increase in the incidence of bloody diarrhea [15].

Complementary food contamination can occur through multiple environmental pathways, including contact with dirty hands, unsafe water, soil, flies, or domestic animals [15–18]. In Bangladesh, Islam et al. (2012) found that only 6–11% of freshly prepared foods (<1 h after cooking) were contaminated with E. coli, compared with 40% of foods stored for 4–5 hours at 25–30 °C[14]. In rural Bangladesh, each 1 log₁₀ increase in E. coli in flies collected from the food preparation area corresponded to a 0.31 log₁₀ increase E. coli in food samples collected from the same area [19]. In an urban slum in Dhaka, rice exposed to fly landings was 5.4 times more likely to be contaminated with E. coli than rice protected with netting, with mean contamination reaching 3.1 × 10³ CFU/g; diarrheagenic E. coli and Shigella genes were detected in 65% of exposed samples, demonstrating that higher fly densities directly increase the risk of diarrheal pathogens in children’s food [17].

Domestic animals, particularly chickens, can also transfer bacteria from their beaks to food [12,16,18,20,21]. In urban Bangladesh pathogenic E. coli, Shigella, Campylobacter, Aeromonas, and rotavirus have all been found in complementary food [14,22,23], with one-third of complementary food positive for E. coli (average 38 MPN/g dry weight) [24]. In rural Bangladesh 46–58% of stored complementary food contained E. coli, with 12–28% exhibiting high contamination (≥100 MPN/dry g) [13,25]; food stored for over four hours was 2.5 times more likely to test positive for E. coli [13].

A critical driver of foodborne pathogen proliferation is prolonged and/or unsafe food storage. Unsafe practices include leaving cooked foods uncovered, storing them at warm ambient temperatures, keeping them in contaminated containers, and failing to reheat to boiling before serving. In peri-urban Mali, complementary food that initially contained no fecal coliforms immediately after cooking became heavily contaminated when stored at 33–37 °C for 7–12 hours. Counts reached as high as 290 thermotolerant coliforms (TTC)/g within six hours, whereas food stored under improved conditions—including covering with a lid, handwashing, dishwashing with soap, and reheating to boiling—had contamination levels as low as 9 TTC/g [26,27]. Likewise, in rural Bangladesh, complementary food stored at 36–42 °C for 4–8 hours showed nearly a tenfold increase in fecal coliforms, rising from 1.84 to 2.66 log₁₀ CFU/g [28].

Despite the protective benefits of covering food, caregivers may not cover food because they worry about trapped steam spoiling food faster and leave freshly cooked, and likely microbiologically safe food uncovered, or partially covered for steam release [21]. Meatsafes, locally available food storage hardware that have walls of wire or mesh, can potentially facilitate immediate safe storage of freshly cooked food (S1 Fig). Meatsafes can also address concerns around trapped steam, since the netted mesh will protect the food even if it is left uncovered inside the meatsafe. In other words, where immediately covering of freshly cooked foods is not possible, meatsafes can provide an alternative method for immediate safe storage.

Prior interventions on food hygiene have relied on behavior-change communication (BCC) intensive strategies. These intensive BCC interventions in Bangladesh, Mali, and Malawi, using repeated household visits, demonstrations, and participatory activities, produced short-term gains in hygiene practices and reductions in contamination and diarrhea [27–29]. However, interventions relying on intensive BCC strategies are not only resource- and labor-intensive and therefore difficult to scale, but also often unsustainable [30] and behaviors revert to baseline levels once promotional activities cease.

In this study, we drew on behavioral economics principles of nudges, subtle environmental cues that guide behavior without explicit instruction and paired one-time BCC with a hardware “nudge” of a meatsafe. Nudges have improved nutrition and hygiene practices in other settings: visual footprints leading to school handwashing stations significantly increased handwashing [31,32] and plates printed with dietary guidance influenced children’s food choices [33].

To assess the impact of meatsafes, along with one-time BCC on proper use and maintenance of meatsafes, and general food safety practices, we conducted a randomized controlled trial (RCT) in urban Dhaka. This study aimed to evaluate their effect on microbial contamination of complementary foods and caregiver-reported diarrhea in children under two.

## Methods

### Study setting and population

Korail, an informal settlement in Dhaka, Bangladesh, packs over 100,000 residents into just 0.4 km², all of whom are served by poor WASH infrastructure, including intermittent piped water and shallow tubewells and shared latrines that often drain into the environment [34]. Households were eligible if 1) they had a child aged 6–24 months; 2) they had been living in Korail for 6 months before the study began and would continue to be available for 6 months from the start of the study; and 3) they owned neither a meatsafe nor a refrigerator. Food samples had to be stored ≥4 hours to be eligible for collection.

### Sample size and power calculation

Our sample size calculations were based on prior findings from Bangladesh that 1) 30.4% of complementary food samples from informal settlements in Dhaka had ≥ 100 CFU *E. coli*/g [35] and 2) in rural areas uncovered foods were twice as contaminated as covered foods (adjusted prevalence ratio (aPR): 2.0, 95% CI: 1.2, 3.2) [13]. Assuming a conservative 50% compliance rate with intervention, we estimated a 25% reduction in contamination prevalence in the intervention arm, from 30.4% to 22.8%. To detect this effect with 80% power and a two-sided α of 0.05, over five repeated food-sampling visits per household and an intracluster correlation coefficient (ICC) of 0.04 [36,37], assuming 15% attrition we estimate a sample size was 145 households per study arm.

### Randomization

**Fig 1:**
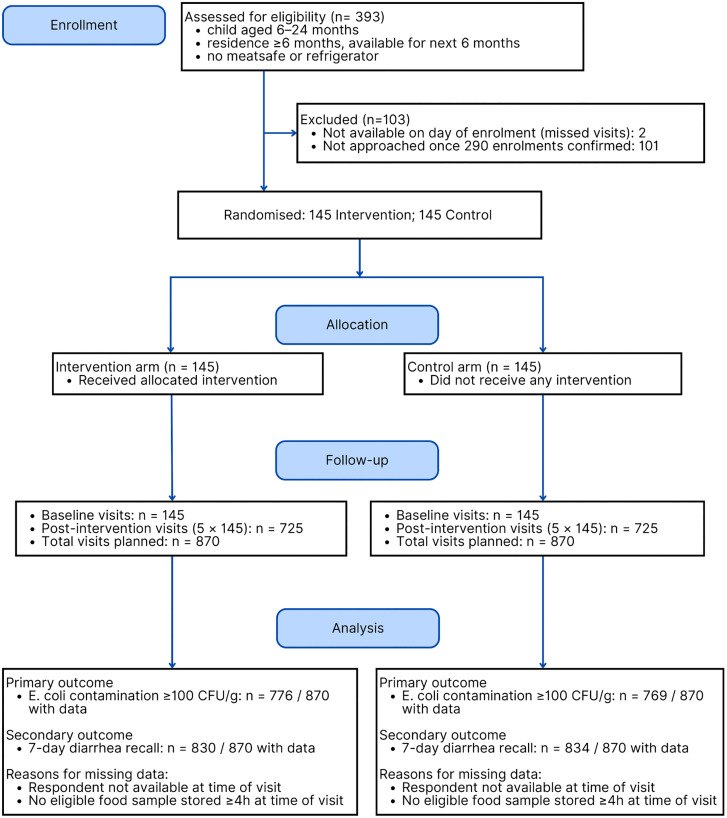
CONSORT 2025 flow diagram of household enrolment, randomisation, allocation, follow-up, and analysis.

After collecting baseline data, a central coordinator randomly generated the allocation sequence using a computer-based random number generator in Stata, with no blocking or stratification. Household IDs were entered sequentially in the order of enrollment, and each was assigned to intervention or control with equal probability. To reduce the risk of contamination between arms, enrolled households were required to be at least 100 meters apart; enumerators moved in a clockwise direction from a fixed starting point in the settlement, knocking on every alternate household, to maintain geographic spacing during recruitment. Allocation was implemented centrally by a coordinator who was not involved in data collection; field enumerators learned a household’s assignment only at the point of intervention delivery, one to two days after the baseline visit. Intervention households received a meatsafe within one to two days of assignment; control households received a meatsafe at the end of the study. Trial enrolment, allocation, follow-up, and analysis are shown in the CONSORT flow diagram (Fig 1).

### Intervention components

**Fig 2:**
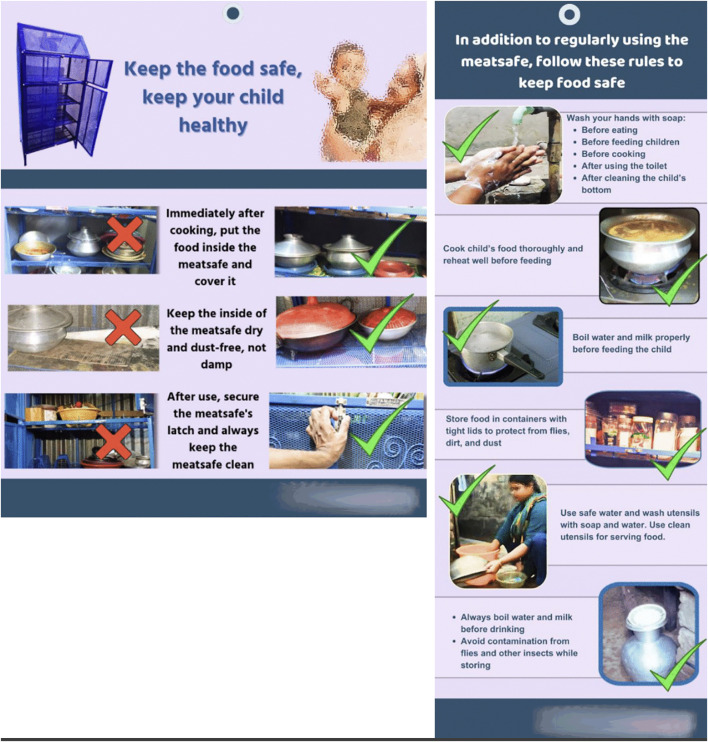
Behavior change communication (BCC) posters.

The intervention consisted of a meatsafe and one-time BCC on food hygiene. Each intervention household received a meatsafe along with two illustrated handouts. The first handout (Fig 2) guided caregivers through proper meatsafe use by showing how to place freshly cooked food straight inside, keep pots covered, wipe shelves dry, and lock the door after every use. The second handout (Fig 2) encouraged the use of five key food-safety behaviors: washing hands with soap at critical moments, cooking and reheating food thoroughly, cleaning containers and utensils with safe water, keeping food, water, and milk tightly covered, and storing them where flies and dust cannot reach [38].

### Data collection

Baseline data collection began on August 11, 2024. Following the intervention, households were revisited every two weeks over a 12-week period, yielding five post-intervention (PI) visits. These subsequent visits are referred to as PI-1 through PI-5, corresponding respectively to the first through fifth post-intervention assessments. The final data collection visit (PI-5) was conducted on November 3, 2024.

### Process outcomes: food storage practices

At every post-intervention visit (PI-1 → PI-5) field research assistants (FRAs) conducted a structured spot-check in each household, noting the food type, the storage setting, and the degree of covering by food type: 1) cooked food (e.g., rice, lentils, curries, and leftovers); 2) raw ingredients (e.g., flour, oil, sugar, and spices); 3) raw fruits/vegetables (e.g., produce ranging from onions and potatoes to bananas and leafy greens); and 4) store-bought foods (e.g., factory-sealed snacks and beverages.

Storage locations included the meatsafe distributed by the project, non-project cabinets other than the meatsafe, including ordinary closed cupboard or wardrobes that offers solid walls but little ventilation and were a pre-existing form of storage in most Korail households (S2 Fig), open shelves (S3 Fig), under the bed, and other areas, including surfaces such as furniture tops. For every food item observed, cover status was classified verbatim as “Present, but no cover” (completely exposed), “Present, but partially covered” (loosely shielded by a cloth, lid, or untied bag), or “Present and fully covered” (fully sealed inside a lidded pot, knotted bag, or screw-top jar).

### Primary outcome: *E. coli* contamination prevalence

Households were informed that follow-up visits would occur every two weeks (typically within 1–2 days of the originally scheduled visit). At each PI visit, FRAs collected a stored samples of the main complementary food prepared for the index child, excluding breastmilk and shelf-stable items. If no sample had been stored ≥4 hours, a follow-up visit was attempted within the next day; otherwise, no sample was collected.

*E. coli* concentration was assessed via culture plating, with biochemical confirmation and enumeration of colony-forming units (CFU) per gram wet weight of food sample [14]. Briefly, 25 g of solid or semi-solid food were mixed with 225 mL of 0.1% peptone water and homogenized using a Stomacher 400 circulator (Seward Co. Ltd., West Sussex, UK) at 230 rpm for one minute [39,40]. If necessary, a 1 mL aliquot of the homogenate was serially diluted (2–5 fold). Dilutions were inoculated onto Tryptone Bile X-glucuronide (TBX) agar (Oxoid, UK) using the pour plate method and incubated at 44 °C for 18–24 hours. Blue-green colonies were considered presumptive *E. coli*, with confirmation via API 20E kits (bioMérieux, Marcy l’Étoile, France). *E. coli* ATCC 25922 and *Klebsiella pneumoniae* ATCC 35657 were used as positive and negative controls, respectively. The limit of detection for this method was 10 CFU *E. coli*/wet g food.

We designated food samples with ≥100 CFU *E. coli*/wet g food as ‘unsafe,’ following the UK’s Public Health Laboratory Service Advisory Committee’s microbiological criteria for ready-to-eat foods, including infant and complementary foods, where this threshold marks unsatisfactory food hygiene and indicates potential health risk [41]. This binary outcome aids public health interpretation and mitigates the influence of skewed data, improving clarity and robustness in regression models.

### Secondary outcome: diarrhea in the past week

During each visit we asked the primary caregiver to recall whether the child had experienced an episode of WHO-defined diarrhea (three or more loose stools within a 24-hour period) in the 7 days preceding each household visit [42].

### Data analysis

The protocol pre-specified intention-to-treat analysis of the binary contamination outcome using regression with clustering at the household level, consistent with the repeated-measures design. We implemented this as a population-averaged modified Poisson regression with robust standard errors clustered at the household level — the preferred estimator for binary outcomes in clustered randomized trials because it directly models prevalence ratios without the convergence instability of log-binomial regression, and the sandwich variance estimator correctly accounts for within-household correlation across repeated food samples without requiring distributional assumptions about the random effects [43,44]:


$$\begin{array}{c} ogE\left[Y_{it}\right]=\beta_{0}+\beta_{1}{Intervention}_{i}+\beta_{2}{Visit}_{t}+\beta_{3}({Intervention}_{i}\times {Visit}_{t})+\gamma ⊤X_{it}+u_{i} \end{array}$$

To separate immediate changes from longer-term patterns, we additionally ran models that isolated the effect of the baseline to first post-intervention (PI-1) change and the subsequent slope from PI-1 to PI-5.

### Covariates

To identify environmental determinants of high-level *E. coli* contamination (≥100 CFU *E. coli*/wet g food) we first ran separate bivariate regressions for each predictor. Robust Poisson models estimated PRs for (i) storage duration (4–8 hours and >8 hours) and (ii) food sample temperature and humidity at collection. Temperature and relative humidity of storage area were modeled jointly, because higher temperature accelerates bacterial metabolism and humidity maintains the surface moisture required for replication, allowing independent assessment of each factor’s effect [45–47]. Variables with *p* ≤ 0.20 in bivariate analyses were then included as covariates in the final model. Analysis was conducted using Stata 18.

### Minimal BCC messages recall

A structured questionnaire assessed BCC message recall among 145 participants. Caregivers viewed three posters and identified the poster they had seen during the BCC explaining how to use meatsafe, then freely recalled hygiene messages. Field staff completed five post-intervention spot-check rounds (PI-1–PI-5; n = 684), documenting meatsafe usage. Data were summarized with descriptive statistics.

### Ethics statement

This study was approved by the Committee for Protection of Human Subjects at the University of California, Berkeley (Protocol #2024-04-17365) and the Ethical and Research Review Committees at icddr,b (PR-24022). Written informed consent was obtained from every participant before enrollment.

### Trial registration

This trial was retrospectively registered with the ClinicalTrials.gov Protocol Registration and Results System (PRS) of the U.S. National Library of Medicine (NCT07332078; Brief Title: Meatsafe Food Storage and Diarrhea Prevention Trial in Urban Bangladesh; Unique Protocol ID: 092000310-UCB-0310). The trial was registered on January 12, 2026, after completion of data collection. Participant enrollment began prior to registration due to administrative delays in registry processing. The study protocol, eligibility criteria, primary and secondary outcomes, and statistical analysis plan were finalized and approved by institutional review boards before the start of data collection, and no changes to prespecified outcomes or analyses were made after trial initiation. The authors confirm that all ongoing and related trials for this intervention are registered. The registry record is publicly accessible at ClinicalTrials.gov (https://clinicaltrials.gov/study/NCT07332078).

## Results

Out of 145 control and 145 intervention households enrolled at baseline, we obtained complementary food samples stored ≥4 hours from 137 control and 136 intervention households. The sample sizes across post-intervention visits (PI-1 to PI-5) ranged from 122 to 131 per arm. Across the six rounds, 1545 complementary food samples (769 control and 776 intervention) had valid *E. coli* results.

### Characteristics of the study population

At baseline, the control and intervention arms were closely matched on most demographic, socioeconomic and WASH variables (Table 1). There was no difference in the average concentration of *E. coli* (1.91 log_10_ vs 1.67 log_10_ CFU *E. coli*/wet g food), prevalence of very high contamination ≥100 CFU *E. coli*/wet g food (49.6% vs 39.0%) and 7-day caregiver-reported diarrhea (9.7% vs 9.0%). However, the proportion of foods with *E. coli* ≥ 10 CFU *E. coli*/wet g food was (63.5%) in controls than in the intervention arm (47.1%) and mothers were respondents in 86.2% of control households but 93.1% of intervention households.

**Table 1:** Baseline household characteristics of participants in randomized controlled trial of meatsafes in Dhaka, Bangladesh.

Characteristic	Control (n = 145)	Intervention (n = 145)	Total (n = 290)	P value
Child age (months), mean (SD)	14.24 (4.56)	14.11 (5.08)	14.18 (4.82)	0.82
Age group, n/N (%)				0.10
6–11 months	46/145 (44.2)	58/145 (55.8)	104/290 (35.9)	
12–17 months	58/145 (58.6)	41/145 (41.4)	99/290 (34.1)	
18–24 months	41/145 (47.1)	46/145 (52.9)	87/290 (30.0)	
Gender, n/N (%)				0.73
Female	73/145 (51.0)	70/145 (49.0)	143/290 (49.3)	
Male	72/145 (49.0)	75/145 (51.0)	147/290 (50.7)	
Household size				
Total people, mean (SD)	4.13 (1.23)	3.99 (1.22)	4.06 (1.23)	0.34
Compound households, mean (SD)	12.30 (8.67)	11.52 (8.70)	11.91 (8.68)	0.45
Rooms, n/N (%)				0.10
1 room	130/145 (89.7)	133/145 (91.7)	263/290 (90.7)	
2 rooms	15/145 (10.3)	8/145 (5.5)	23/290 (7.9)	
≥3 rooms	0/145 (0.0)	4/145 (2.8)	4/290 (1.4)	
Monthly income (BDT), mean (SD)	16,667.6 (6,213.4)	16,675.9 (6,019.2)	16,671.7 (6,115.0)	0.99
Food expenditure (BDT)				
Yesterday	279.8 (230.1)	294.2 (226.9)	287.0 (228.5)	0.59
Past week	1,784.8 (1,137.2)	1,840.1 (1,017.6)	1,812.5 (1,078.6)	0.66
Snacks past week	418.7 (367.4)	410.4 (379.6)	414.6 (373.3)	0.85
Electricity, n/N (%)				0.30
Always	100/145 (69.0)	108/145 (74.5)	208/290 (71.7)	
Most of the time	45/145 (31.0)	37/145 (25.5)	82/290 (28.3)	
Internet access, n/N (%)	31/145 (21.4)	35/145 (24.1)	66/290 (22.8)	0.58
Color TV ownership, n/N (%)	23/145 (15.9)	23/145 (15.9)	46/290 (15.9)	1.00
Households with ≥1 phone, n/N (%)	133/145 (91.7)	136/145 (93.8)	269/290 (92.8)	—
Smartphones, n/N (%)				0.42
0	67/145 (46.2)	53/145 (36.6)	120/290 (41.4)	
1	62/145 (42.8)	75/145 (51.7)	137/290 (47.2)	
≥2	16/145 (11.0)	17/145 (11.7)	33/290 (11.4)	
Caregivers, n/N (%)				0.46
1	104/145 (71.7)	113/145 (77.9)	217/290 (74.8)	
≥2	41/145 (28.3)	32/145 (22.1)	73/290 (25.2)	
Primary caregiver, n/N (%)				0.11
Mother	125/145 (86.2)	135/145 (93.1)	260/290 (89.7)	
Other relative	20/145 (13.8)	10/145 (6.9)	30/290 (10.3)	
Caregiver age (years), mean (SD)	26.83 (9.63)	24.40 (6.86)	25.61 (8.40)	0.01
Caregiver education (years), mean (SD)	5.80 (3.26)	5.99 (3.11)	5.90 (3.18)	0.62
Food preparation area, n/N (%)				0.01
Same room	113/145 (77.9)	128/145 (88.3)	241/290 (83.1)	
Separate/other	32/145 (22.1)	17/145 (11.7)	49/290 (16.9)	
Toilet type, n/N (%)				0.16
Sanitary latrine	105/145 (72.4)	118/145 (81.4)	223/290 (76.9)	
Pit latrine/other	40/145 (27.6)	27/145 (18.6)	67/290 (23.1)	
Housing material, n/N (%)				
Roof tin	106/145 (73.1)	112/145 (77.2)	218/290 (75.2)	0.61
Wall tin	93/145 (64.1)	100/145 (69.0)	193/290 (66.6)	0.44
Floor cement/brick	136/145 (93.8)	135/145 (93.1)	271/290 (93.4)	0.81
Cooking fuel, n/N (%)				0.34
Natural gas	132/145 (91.0)	137/145 (94.5)	269/290 (92.8)	
LPG/wood	13/145 (9.0)	8/145 (5.5)	21/290 (7.2)	
Water sources, n/N (%)				0.53
1 source	133/145 (91.7)	129/145 (89.0)	262/290 (90.3)	
≥2 sources	12/145 (8.3)	15/145 (11.0)	27/290 (9.7)	
Primary source, n/N (%)				0.17
WASA + tubewell	100/145 (69.0)	84/145 (57.9)	184/290 (63.4)	
Piped yard/tap/other	45/145 (31.0)	61/145 (42.1)	106/290 (36.6)	
Drinking water treated, n/N (%)	31/144 (21.5)	25/145 (17.2)	56/289 (19.4)	0.36
Food storage area conditions				
Temperature (°C), mean (SD)	30.30 (2.00)	29.87 (1.88)	30.09 (1.94)	0.06
Humidity (%), mean (SD)	79.91 (7.06)	81.42 (6.59)	80.66 (6.85)	0.08
Stored food temp (°C), mean (SD)	30.83 (1.91)	30.60 (1.99)	30.71 (1.95)	0.33
E. coli log₁₀ CFU/g, mean (SD)	1.91 (1.77)	1.67 (2.08)	1.79 (1.93)	0.31
E. coli ≥10 CFU/g, n/N (%)	87/137 (63.5)	64/136 (47.1)	151/273 (55.3)	0.01
E. coli ≥100 CFU/g, n/N (%)	68/137 (49.6)	53/136 (39.0)	121/273 (44.3)	0.08
Child diarrhea, n/N (%)				
7-day recall	14/145 (9.7)	13/145 (9.0)	27/290 (9.3)	0.84

Data are mean (SD) or n/N (%). P values from t tests (continuous) or χ² tests (categorical).

BDT = Bangladeshi Taka (~119 BDT = 1 USD in 2024)

### Trends of *E. coli* contamination and diarrhea

**Fig 3:**
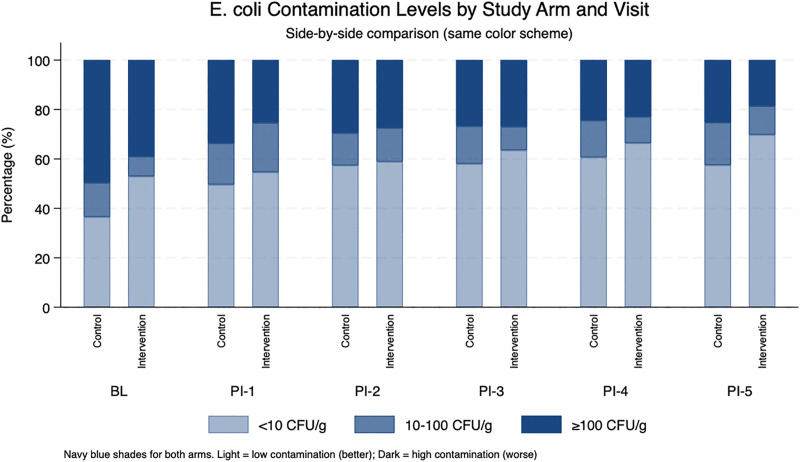
*E. coli* contamination of stored complementary foods by study arm and visit.

*E. coli* contamination ≥100 CFU *E. coli*/wet g food decreased in both study arms, from 49.6% to 25.2% in the control arm and 39.0% to 18.6% in the intervention arm (Fig 3 and S1 Table).

**Fig 4:**
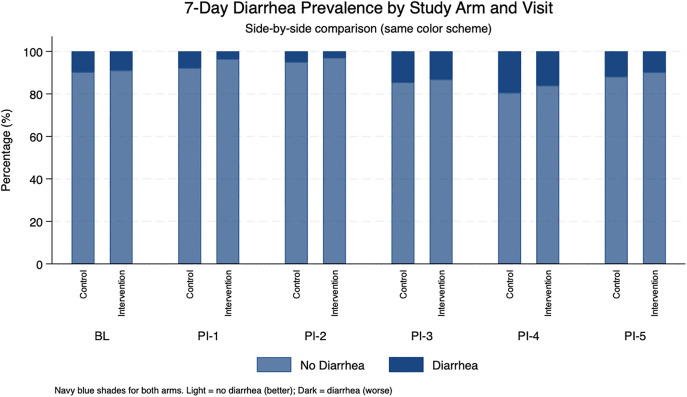
Caregiver-reported child diarrhea (7-day recall), by study arm and visit.

**Fig 5:**
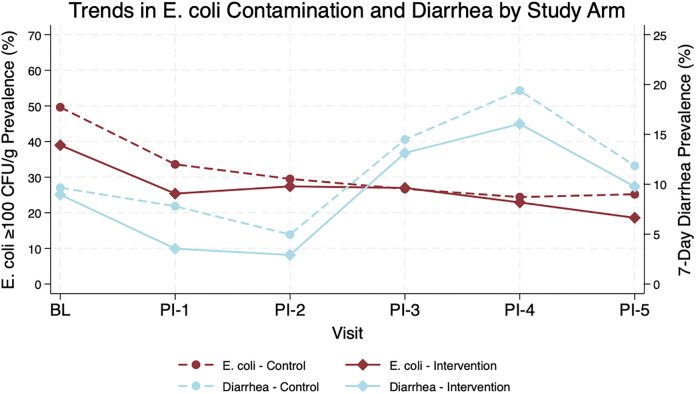
Combined trends in contamination and diarrhea outcomes, by study arm.

Diarrhea prevalence fluctuated, decreasing from baseline (9.7% control, 9.0% intervention) to PI-2 (5.0% control, 2.9% intervention), then peaking at PI-4 (19.4% control, 16.1% intervention) (Fig 4 and S2 Table). The combined trajectories of food contamination and diarrhea outcomes, plotted together by study arm across all visits, are shown in Fig 5.

### Food storage practices

In the intervention arm, almost all cooked food was stored in a meatsafe (87.7% at PI-1, rising to 99.2% at PI-5), whereas control households mainly (56.1–65.4%) stored food on shelves and, secondarily (16.0–23.0%) under beds and in non-project cabinets (5.5-12.2%) (S3 Table).

Cooked dishes placed in a meatsafe in the intervention arm were almost always fully covered (66.9% at PI-1 to 88.4% at PI-5). In the control arm, cooked food stored on open shelves fully covered 47.1% to 64.3% of the time. Raw fruits and vegetables placed under beds remained uncovered in both arms: 90.0% (intervention) and 73.2% (control) at PI-1; and 47.2% and 56.0% at PI-5, respectively. By PI-5, 78% of intervention households stored raw ingredients fully covered inside meatsafe, while in the controls, only about 25% were fully covered and roughly half were uncovered (S4 Table).

### Association between environmental conditions and food contamination

In bivariate analyses, storing food for >8 h was associated with a higher prevalence of high-level contamination by 66% (PR = 1.66 [1.37, 2.02]) so was included as a covariate in the final model (Table 2). Food temperature at the time of sampling was not associated with contamination (PR = 0.99 [0.94, 1.05]) and was excluded from subsequent models. In a joint model, storage area temperature remained non-significant (PR = 1.00 [0.95, 1.06]), but each 1% increase in the relative humidity of the storage area was associated with a slight increase in the prevalence of contamination (PR = 1.01 [1.00, 1.02]). Based on the predefined threshold of p ≤ 0.20, storage duration, storage area temperature, and storage area humidity were retained as covariates in the final multivariate model.

**Table 2:** Environmental determinants of *E. coli* ≥100 CFU *E. coli*/wet g food (post-intervention visits).

Determinant	PR (95% CI)	P value	N (obs)	N (hh)
Storage duration				
>8 hours vs ≤ 8 hours	1.66 (1.37–2.02)***	<0.001	1,272	285
Food sample temperature				
Per 1°C increase	0.99 (0.94–1.05)	0.824	1,266	285
Storage area conditions (joint model)				
Temperature (°C)	1.00 (0.95–1.06)	0.890	1,268	286
Humidity (%)	1.01 (1.00–1.02)	0.177	1,268	286

PR: Prevalence Ratio from Poisson regression with robust standard errors

*Joint Model

### Impact of the intervention on high *E. coli* contamination

There was no association between the meatsafe and the prevalence of high E. coli contamination (≥100 CFU *E. coli*/wet g) (aPR = 0.83 [0.55, 1.26]) (Table 3). There was no change in the prevalence of contamination from PI-1 to PI-5 in either arm (aPR = 0.93; 95% CI: 0.85–1.02). In the multivariate analysis including variables to represent intervention arm and study visit, storing food for more than eight hours remained strongly associated with a higher prevalence of contamination (aPR = 1.49; 95% CI: 1.20–1.86), while storage area temperature (aPR = 0.99; 95% CI: 0.94–1.05) and humidity (aPR = 1.00; 95% CI: 0.99–1.01) were not significantly associated with contamination.

**Table 3:** Intention-to-treat Poisson model predicting high-level *E. coli* contamination (≥ 100 CFU *E. coli*/wet g food).

Variable	Main effect (PI-1 to PI-5) PR (95% CI)	Baseline to PI-1 PR (95% CI)
Intervention arm	0.83 (0.55–1.26)	0.74 (0.54–1.01)
Time effect		
Post-intervention visit (per +1)	0.93 (0.85–1.02)	—
PI-1 vs Baseline	—	0.75 (0.55–1.01)
Interaction terms		
Intervention × Visit	1.02 (0.90–1.16)	—
Intervention × PI-1	—	1.01 (0.64–1.61)
Covariates		
Storage area temperature (°C)	0.99 (0.94–1.05)	0.93 (0.85–1.02)
Storage area humidity (%)	1.00 (0.99–1.01)	1.00 (0.98–1.02)
Storage ≥8 hours	1.49 (1.20–1.86)***	1.20 (0.93–1.54)
Model statistics		
N (observations)	1,268	493
N (households)	286	285
Wald χ²	22.30***	18.85**
Interaction χ² (p-value)	0.09 (0.77)	0.00 (0.96)

*PR = Prevalence ratio. Models estimated with modified Poisson regression and robust SEs clustered by household. Models adjust for food storage temperature, humidity, and storage > 8h. *p < 0.05, *p < 0.01, *p < 0.001.

In both arms, contamination fell by ~25% from baseline to the first post-intervention visit (aPR = 0.75; 95% CI: 0.55–1.01), and the magnitude of this initial drop did not differ by arm (interaction aPR = 1.01; 95% CI: 0.64–1.61).

### Effect of the intervention on diarrhea

There was no association between the meatsafe and caregiver-reported diarrhea prevalence (PR = 0.56 [0.24, 1.31]) (Table 4). From PI-1 to PI-5, diarrhea prevalence increased by ~24% per visit (PR = 1.24; 95% CI: 1.06–1.45), with no difference between arms (interaction PR = 1.10; 95% CI: 0.89–1.36). In the multivariate analysis including variables to represent intervention arm and study visit, storage area temperature (PR = 1.03; 95% CI: 0.94–1.12) and humidity (PR = 1.01; 95% CI: 0.99–1.03) were not significantly associated with diarrhea, and storing food for more than eight hours was also not significantly associated (PR = 0.81; 95% CI: 0.54–1.20).

**Table 4:** Poisson model predicting caregiver-reported diarrhea (7-day recall).

Variable	Main effect (PI-1 to PI-5) PR (95% CI)	Baseline to PI-1 PR (95% CI)
Intervention arm	0.56 (0.24–1.31)	0.85 (0.33–2.18)
Time effect		
Post-intervention visit (per +1)	1.24 (1.06–1.45)**	—
PI-1 vs Baseline	—	1.11 (0.45–2.74)
Interaction terms		
Intervention × Visit	1.10 (0.89–1.36)	—
Intervention × PI-1	—	0.61 (0.15–2.46)
Covariates		
Storage area temperature (°C)	1.03 (0.94–1.12)	0.88 (0.67–1.14)
Storage area humidity (%)	1.01 (0.99–1.03)	0.95 (0.90–1.01)
Storage ≥8 hours	0.81 (0.54–1.20)	1.88 (0.94–3.77)
Model statistics		
N (observations)	1,268	493
N (households)	286	285
Wald χ²	23.50***	10.27
Interaction χ² (p-value)	0.71 (0.40)	0.48 (0.49)

*PR = Prevalence ratio. Models estimated with modified Poisson regression and robust standard errors clustered by household. Models adjust for food storage temperature, humidity, and storage > 8h. *p < 0.05, *p < 0.01, *p < 0.001.

From baseline to PI-1, diarrhea prevalence did not change significantly (PR = 1.11; 95% CI: 0.45–2.74) in both arms (interaction PR = 0.61; 95% CI: 0.15–2.46).

### Minimal BCC message recall findings

Recall of the three key meatsafe usage and maintenance messages was high, with 63–88% of respondents able to state them correctly (Table 5). Specifically, 88.3% recalled the recommendation to place food inside the meatsafe and cover it immediately after cooking, 76.7% remembered to secure the latch and keep the device clean, and 63.3% mentioned keeping the interior dry and dust-free.

**Table 5:** Message recalled from Meatsafe usage and maintenance BCC and general food hygiene recommendation BCC.

Message recalled	n/N (%)
Meatsafe usage and maintenance BCC	
Immediately after cooking, put the food inside the meatsafe and cover it	106/120 (88.3)
Keep the inside of the meatsafe dry and dust-free, not damp	76/120 (63.3)
After use, secure the meatsafe’s latch and always keep it clean	92/120 (76.7)
General food hygiene recommendation BCC	
Wash hands with soap before eating	85/120 (70.8)
Wash hands with soap before feeding children	74/120 (61.7)
Wash hands with soap before cooking	52/120 (43.3)
Wash hands with soap after using the toilet	80/120 (66.7)
Wash hands with soap after cleaning the child’s bottom	64/120 (53.3)
Cook child’s food thoroughly and reheat well before feeding	21/120 (17.5)
Use safe water and wash utensils with soap and water; use clean utensils for serving food	9/120 (7.5)
Store food in containers with tight lids to protect from flies, dirt, and dust	4/120 (3.3)
Always boil water and milk before drinking	8/120 (6.7)

In contrast, recall of general food hygiene recommendations was more variable and generally lower. Among handwashing messages, 70.8% recalled washing hands before eating, 66.7% after toilet use, 61.7% before feeding children, 53.3% after cleaning a child’s bottom, and 43.3% before cooking. Messages related to food preparation and storage were least frequently remembered: only 17.5% recalled thorough cooking and reheating of food, 7.5% mentioned use of safe water and clean utensils, 6.7% recalled boiling water or milk before consumption, and just 3.3% remembered storing food in tight-lidded containers.

## Discussion

This RCT evaluated whether providing a low-cost, food cabinet with mesh walls (“meatsafe”), paired with a one-time BCC, reduced fecal contamination of children’s complementary foods and caregiver-reported diarrhea in an informal settlement with poor WASH infrastructure in Korail, Dhaka. After adjusting for environmental factors most strongly linked with contamination, we found that the intervention had no effect on proportion of foods exceeding 100 CFU E. coli/wet g food or on caregiver-reported seven-day diarrhea prevalence among children <2 years old.

Several mechanisms may explain the null findings. Firstly, the design limitations of the meatsafe itself may have constrained its protective effect. The mesh-walled meatsafe may have blocked flies, but not other sources of contamination and it did nothing to slow down the exponential growth of any microbial contamination present in the food before it was stored in the meatsafe. In an experimental study by Lindeberg et al. (2018) in a Dhaka slum, rice containers left uncovered were more than five times as likely to contain E. coli compared to net-covered controls. However, even among the net-covered controls where no flies landed, 28% were still contaminated, suggesting that contamination may occur via airborne particles, regurgitation or defecation through mesh, or other environmental sources [17].

Secondly, the effect of being observed and being asked questions about food handling and hygiene, and collecting food samples from the households every two weeks, may have prompted unmeasured improvements in hygiene behaviors not measured directly, such as handwashing before food preparation, in both the arms. This phenomenon of measurement-induced behavior change has strong precedent in similar trials. The WASH Benefits Kenya study found that no intervention outperformed their active control arm, suggesting that regular monitoring visits alone may drive hygiene improvements [4]. The SHINE trial in Zimbabwe documented that hygiene practices peaked during observation visits then waned between them, demonstrating the temporary nature of such reactive behaviors [6]. Our biweekly visit schedule may have been frequent enough to sustain these temporary improvements throughout the study period, potentially masking the true effects of the meatsafe intervention.

Finally, given the low recall of broader hygiene practices in our results, minimal BCC or nudge-like strategies alone may prove to be too low on intensity to affect food contamination outcomes in resource-constraint household environments. Minimal BCC was designed, drawing on behavioral economics principles of nudges, as a response to prior intensive interventions that, while effective in the short term, were resource- and labor-intensive, difficult to scale, and unsustainable once promoter activities ended [30]. Past food hygiene interventions that achieved impact include the Banja la Ukhondo program in Malawi, which delivered 33 participatory BCC sessions over 31 weeks and reduced self-reported diarrhea by 13 percentage points without providing hardware [29,48,49], and a daily home-visit, intensive BCC-only intervention that virtually eliminated fecal pathogens in weaning foods, with gains persisting for three months in Bangladesh and Mali [26,28]. More robust or sustained communication may therefore be required to reinforce food hygiene behaviors beyond device-specific use.

### Limitations

Given the large decrease in the prevalence of high-level food contamination and the large increase in food covering among both intervention and control households from the baseline to first post-intervention visit, it appears that the results were strongly affected by reactivity to observation.

A second limitation is the use of *E. coli* as an indicator organism. While widely recognized as a marker of fecal contamination [50], most *E. coli* strains are nonpathogenic. Only a subset—such as enterotoxigenic (ETEC), enteropathogenic (EPEC), and enterohemorrhagic (EHEC)—harbor virulence factors capable of causing diarrhea. These diarrheagenic strains may persist at low, undetected levels even when total *E. coli* counts decline, meaning our outcome may underestimate true health risk.

## Conclusion

In this randomized trial, providing a low-cost meatsafe with one-time BCC did not reduce the prevalence of high levels of fecal contamination of stored complementary food or caregiver-reported diarrhea among children <2 years old, despite high levels of meatsafe usage. By demonstrating both the feasibility of household uptake and the limits of its protective benefit, this study suggests that simple, passive technologies alone may be insufficient.

Future research could explore whether pairing more intensive and sustained BCC with affordable thermal technologies such as solar refrigerators, direct-drive fridges, phase-change–salt cookers, and hybrid solar–LPG stoves [51–54]yields greater reductions in food contamination and diarrhea in similar settings, and whether such combinations are feasible and scalable in low-income urban contexts.

## Supporting information

S1 Fig Meatsafe of the type distributed to intervention households. (TIF)

S2 Fig Examples of pre-existing non-project cabinets for food storage. (TIF)

S3 Fig Shelves that are open, and not protected in any way other than being elevated surfaces. (TIF)

S1 Table *E. coli* contamination levels by study arm and visit. (DOCX)

S2 Table 7-Day diarrhea prevalence by study arm and visit. (DOCX)

S3 Table Type of food and storage locations across the intervention and control arms at each post-intervention visits. (DOCX)

S4 Table Type of food and cover status in different locations across intervention and control at each post-intervention visit. (DOCX)
