# Supplementary material for: Evaluating the impact of a low-cost food storage intervention on complementary food contamination and diarrheal disease in low-income urban households: A randomized controlled trial in Dhaka, Bangladesh
Source: PLOS Glob Public Health. 2026 Jun 11;6(6):e0005883. doi: 10.1371/journal.pgph.0005883 (PMC13257995; doi:10.1371/journal.pgph.0005883)
Supplement: S1 Table — (DOCX) [file pgph.0005883.s004.docx]

*S1 Table. E. coli* contamination levels by study arm and visit

|  | BL | PI-1 | PI-2 | PI-3 | PI-4 | PI-5 |
| --- | --- | --- | --- | --- | --- | --- |
| Control Arm |  |  |  |  |  |  |
| <10 CFU *E. coli*/wet g food | 50 (36.5%) | 62 (49.6%) | 70 (57.4%) | 76 (58.0%) | 77 (60.6%) | 73 (57.5%) |
| 10-100 CFU *E. coli*/wet g food | 19 (13.9%) | 21 (16.8%) | 16 (13.1%) | 20 (15.3%) | 19 (15.0%) | 22 (17.3%) |
| ≥100 CFU *E. coli*/wet g food | 68 (49.6%) | 42 (33.6%) | 36 (29.5%) | 35 (26.7%) | 31 (24.4%) | 32 (25.2%) |
| Total | 137 | 125 | 122 | 131 | 127 | 127 |
| Intervention Arm |  |  |  |  |  |  |
| <10 CFU *E. coli*/wet g food | 72 (52.9%) | 71 (54.6%) | 73 (58.9%) | 80 (63.5%) | 87 (66.4%) | 90 (69.8%) |
| 10-100 CFU *E. coli*/wet g food | 11 (8.1%) | 26 (20.0%) | 17 (13.7%) | 12 (9.5%) | 14 (10.7%) | 15 (11.6%) |
| ≥100 CFU *E. coli*/wet g food | 53 (39.0%) | 33 (25.4%) | 34 (27.4%) | 34 (27.0%) | 30 (22.9%) | 24 (18.6%) |
| Total | 136 | 130 | 124 | 126 | 131 | 129 |

Note: Values represent frequency counts with column percentages. Each column summarizes *E. coli* contamination levels among food samples collected at a specific visit by study arm. BL = Baseline, PI = post-intervention.
