# Supplementary material for: Evaluating the impact of a low-cost food storage intervention on complementary food contamination and diarrheal disease in low-income urban households: A randomized controlled trial in Dhaka, Bangladesh
Source: PLOS Glob Public Health. 2026 Jun 11;6(6):e0005883. doi: 10.1371/journal.pgph.0005883 (PMC13257995; doi:10.1371/journal.pgph.0005883)
Supplement: S2 Table — (DOCX) [file pgph.0005883.s005.docx]

S2 Table. 7-Day diarrhea prevalence by study arm and visit

|  | BL | PI-1 | PI-2 | PI-3 | PI-4 | PI-5 |
| --- | --- | --- | --- | --- | --- | --- |
| Control |  |  |  |  |  |  |
| No diarrhea | 131 (90.3%) | 130 (92.2%) | 134 (95.0%) | 118 (85.5%) | 108 (80.6%) | 119 (88.1%) |
| Diarrhea | 14 (9.7%) | 11 (7.8%) | 7 (5.0%) | 20 (14.5%) | 26 (19.4%) | 16 (11.9%) |
| Total | 145 | 141 | 141 | 138 | 134 | 135 |

Intervention Arm

| No diarrhea | 132 (91.0%) | 136 (96.5%) | 133 (97.1%) | 119 (86.9%) | 115 (83.9%) | 120 (90.2%) |
| --- | --- | --- | --- | --- | --- | --- |
| Diarrhea | 13 (9.0%) | 5 (3.5%) | 4 (2.9%) | 18 (13.1%) | 22 (16.1%) | 13 (9.8%) |
| Total | 145 | 141 | 137 | 137 | 137 | 133 |

Note: Values represent frequency counts with column percentages. Each column
