# Supplementary material for: Evaluating the impact of a low-cost food storage intervention on complementary food contamination and diarrheal disease in low-income urban households: A randomized controlled trial in Dhaka, Bangladesh
Source: PLOS Glob Public Health. 2026 Jun 11;6(6):e0005883. doi: 10.1371/journal.pgph.0005883 (PMC13257995; doi:10.1371/journal.pgph.0005883)
Supplement: S3 Table — (DOCX) [file pgph.0005883.s006.docx]

S3 Table. Type of food and storage locations across the intervention and control arms at each post-intervention visits

|  | Intervention | | | | | Control | | | | |
| --- | --- | --- | --- | --- | --- | --- | --- | --- | --- | --- |
|  | PI-1 | PI-2 | PI-3 | PI-4 | PI-5 | PI-1 | PI-2 | PI-3 | PI-4 | PI-5 |
| **Meatsafe** |  |  |  |  |  |  |  |  |  |  |
| Cooked Food | 114 (87.7%) | 112 (89.6%) | 120 (95.2%) | 129 (97.7%) | 128 (99.2%) |  |  |  |  |  |
| Raw Ingredients | 30 (23.1%) | 41 (32.8%) | 37 (29.4%) | 51 (38.6%) | 48 (37.2%) |  |  |  |  |  |
| Raw Fruits/Vegetables | 34 (26.2%) | 30 (24%) | 28 (22.2%) | 38 (28.8%) | 23 (17.8%) |  |  |  |  |  |
| Store Bought Foods | 73 (56.2%) | 78 (62.4%) | 81 (64.3%) | 83 (62.9%) | 68 (52.7%) |  |  |  |  |  |
| **Non-project cabinet** |  |  |  |  |  |  |  |  |  |  |
| Cooked Food | 1 (0.8%) | 1 (0.8%) | 1 (0.8%) | - | - | 6 (4.8%) | 15 (12.2%) | 14 (10.7%) | 10 (7.9%) | 7 (5.5%) |
| Raw Ingredients | 4 (3.1%) | 1 (0.8%) | 2 (1.6%) | - | 1 (0.8%) | 6 (4.8%) | 7 (5.7%) | 7 (5.3%) | 2 (1.6%) | 3 (2.4%) |
| Raw Fruits/Vegetables | 2 (1.5%) | 1 (0.8%) | 3 (2.4%) | - | 2 (1.6%) | 3 (2.4%) | 7 (5.7%) | 6 (4.6%) | 1 (0.8%) | 1 (0.8%) |
| Store Bought Foods | 1 (0.8%) | - | - | - | 1 (0.8%) | 7 (5.6%) | 12 (9.8%) | 11 (8.4%) | 5 (3.9%) | 7 (5.5%) |
| **Open shelf** |  |  |  |  |  |  |  |  |  |  |
| Cooked Food | 10 (7.7%) | 7 (5.6%) | - | 1 (0.8%) | 1 (0.8%) | 81 (64.8%) | 69 (56.1%) | 82 (62.6%) | 73 (57.5%) | 83 (65.4%) |
| Raw Ingredients | 18 (13.8%) | 17 (13.6%) | 15 (11.9%) | 16 (12.1%) | 16 (12.4%) | 29 (23.2%) | 23 (18.7%) | 28 (21.4%) | 36 (28.3%) | 36 (28.3%) |
| Raw Fruits/Vegetables | 19 (14.6%) | 16 (12.8%) | 12 (9.5%) | 13 (9.8%) | 14 (10.9%) | 30 (24%) | 16 (13%) | 25 (19.1%) | 26 (20.5%) | 15 (11.8%) |
| Store Bought Foods | 7 (5.4%) | 3 (2.4%) | 2 (1.6%) | 3 (2.3%) | 1 (0.8%) | 32 (25.6%) | 28 (22.8%) | 41 (31.3%) | 35 (27.6%) | 31 (24.4%) |
| **Under bed** |  |  |  |  |  |  |  |  |  |  |
| Cooked Food | 2 (1.5%) | 1 (0.8%) | 2 (1.6%) | - | - | 23 (18.4%) | 23 (18.7%) | 21 (16%) | 29 (22.8%) | 23 (18.1%) |
| Raw Ingredients | 3 (2.3%) | 3 (2.4%) | 4 (3.2%) | 4 (3%) | 5 (3.9%) | 10 (8%) | 14 (11.4%) | 10 (7.6%) | 16 (12.6%) | 9 (7.1%) |
| Raw Fruits/Vegetables | 2 (1.5%) | 3 (2.4%) | 5 (4%) | 2 (1.5%) | 2 (1.6%) | 8 (6.4%) | 17 (13.8%) | 11 (8.4%) | 15 (11.8%) | 10 (7.9%) |
| Store Bought Foods | 3 (2.3%) | 2 (1.6%) | - | - | - | 6 (4.8%) | 13 (10.6%) | 7 (5.3%) | 9 (7.1%) | 6 (4.7%) |
| **Other Open Area** |  |  |  |  |  |  |  |  |  |  |
| Cooked Food | 1 (0.8%) | 3 (2.4%) | 2 (1.6%) | - | - | 15 (12%) | 11 (8.9%) | 8 (6.1%) | 6 (4.7%) | 7 (5.5%) |
| Raw Ingredients | 23 (17.7%) | 27 (21.6%) | 31 (24.6%) | 41 (31.1%) | 38 (29.5%) | 31 (24.8%) | 36 (29.3%) | 46 (35.1%) | 47 (37%) | 49 (38.6%) |
| Raw Fruits/Vegetables | 29 (22.3%) | 28 (22.4%) | 29 (23%) | 35 (26.5%) | 35 (27.1%) | 41 (32.8%) | 39 (31.7%) | 46 (35.1%) | 40 (31.5%) | 49 (38.6%) |
| Store Bought Foods | 1 (0.8%) | 3 (2.4%) | 2 (1.6%) | - | - | 6 (4.8%) | 8 (6.5%) | 9 (6.9%) | 4 (3.1%) | 3 (2.4%) |
