# Supplementary material for: Evaluating the impact of a low-cost food storage intervention on complementary food contamination and diarrheal disease in low-income urban households: A randomized controlled trial in Dhaka, Bangladesh
Source: PLOS Glob Public Health. 2026 Jun 11;6(6):e0005883. doi: 10.1371/journal.pgph.0005883 (PMC13257995; doi:10.1371/journal.pgph.0005883)
Supplement: S4 Table — (DOCX) [file pgph.0005883.s007.docx]

S4 Table. Type of food and cover status in different locations across intervention and control at each post-intervention visit

|  | Intervention | | | | | | Control | | | | |
| --- | --- | --- | --- | --- | --- | --- | --- | --- | --- | --- | --- |
|  | PI-1 | PI-2 | PI-3 | PI-4 | PI-5 | PI-1 | | PI-2 | PI-3 | PI-4 | PI-5 |
| **Project Meatsafe – Cooked Foods** |  |  |  |  |  |  | |  |  |  |  |
| Present, but no cover | 8 (6.3%) | 5 (4.1%) | 2 (1.6%) | 1 (0.8%) | 3 (2.3%) |  | |  |  |  |  |
| Present, but partially covered | 34 (26.8%) | 27 (22.0%) | 16 (12.9%) | 17 (13.1%) | 12 (9.3%) |  | |  |  |  |  |
| Present and fully covered | 85 (66.9%) | 91 (74.0%) | 106 (85.5%) | 112 (86.2%) | 114 (88.4%) |  | |  |  |  |  |
| Project Meatsafe – Raw Ingredients |  |  |  |  |  |  | |  |  |  |  |
| Present, but no cover | 18 (47.4%) | 24 (43.6%) | 25 (46.3%) | 26 (30.2%) | 14 (17.9%) |  | |  |  |  |  |
| Present, but partially covered | 3 (7.9%) | 3 (5.5%) | 0 (0.0%) | 1 (1.2%) | 3 (3.8%) |  | |  |  |  |  |
| Present and fully covered | 17 (44.7%) | 28 (50.9%) | 29 (53.7%) | 59 (68.6%) | 61 (78.2%) |  | |  |  |  |  |
| **Project Meatsafe – Raw Fruits/Vegetables** |  |  |  |  |  |  | |  |  |  |  |
| Present, but no cover | 29 (80.6%) | 18 (54.5%) | 13 (44.8%) | 21 (52.5%) | 13 (56.5%) |  | |  |  |  |  |
| Present, but partially covered | 4 (11.1%) | 3 (9.1%) | 1 (3.4%) | 1 (2.5%) | 4 (17.4%) |  | |  |  |  |  |
| Present and fully covered | 3 (8.3%) | 12 (36.4%) | 15 (51.7%) | 18 (45.0%) | 6 (26.1%) |  | |  |  |  |  |
| **Project Meatsafe – Store Packaged Bought Foods** |  |  |  |  |  |  | |  |  |  |  |
| Present, but no cover | 29 (35.8%) | 13 (15.9%) | 14 (16.9%) | 20 (23.8%) | 11 (15.9%) |  | |  |  |  |  |
| Present, but partially covered | 2 (2.5%) | 1 (1.2%) | 1 (1.2%) | 1 (1.2%) | 0 (0.0%) |  | |  |  |  |  |
| Present and fully covered | 50 (61.7%) | 68 (82.9%) | 68 (81.9%) | 63 (75.0%) | 58 (84.1%) |  | |  |  |  |  |
| **Non-project cabinet – Cooked Food** |  |  |  |  |  |  | |  |  |  |  |
| Present, but no cover | 0 (0.0%) | 0 (0.0%) | 0 (0.0%) |  |  | 1 (7.7%) | | 0 (0.0%) | 1 (7.1%) | 0 (0.0%) | 2 (28.6%) |
| Present, but partially covered | 0 (0.0%) | 1 (100.0%) | 1 (100.0%) |  |  | 5 (38.5%) | | 3 (18.8%) | 4 (28.6%) | 4 (40.0%) | 3 (42.9%) |
| Present and fully covered | 2 (100.0%) | 0 (0.0%) | 0 (0.0%) |  |  | 7 (53.8%) | | 13 (81.3%) | 9 (64.3%) | 6 (60.0%) | 2 (28.6%) |
| **Non-project cabinet – Raw Ingredients** |  |  |  |  |  |  | |  |  |  |  |
| Present, but no cover | 3 (75.0%) | 1 (100.0%) | 2 (100.0%) |  | 0 (0.0%) | 4 (57.1%) | | 10 (83.3%) | 6 (66.7%) | 1 (20.0%) | 1 (25.0%) |
| Present, but partially covered | 0 (0.0%) | 0 (0.0%) | 0 (0.0%) |  | 0 (0.0%) | 2 (28.6%) | | 0 (0.0%) | 0 (0.0%) | 1 (20.0%) | 0 (0.0%) |
| Present and fully covered | 1 (25.0%) | 0 (0.0%) | 0 (0.0%) |  | 1 (100.0%) | 1 (14.3%) | | 2 (16.7%) | 3 (33.3%) | 3 (60.0%) | 3 (75.0%) |
| **Non-project cabinet – Raw Fruits/Vegetables** |  |  |  |  |  |  | |  |  |  |  |
| Present, but no cover | 2 (100.0%) | 2 (100.0%) | 4 (100.0%) |  | 1 (50.0%) | 5 (100.0%) | | 7 (77.8%) | 6 (100.0%) | 0 (0.0%) | 1 (100.0%) |
| Present, but partially covered | 0 (0.0%) | 0 (0.0%) | 0 (0.0%) |  | 0 (0.0%) | 0 (0.0%) | | 0 (0.0%) | 0 (0.0%) | 1 (100.0%) | 0 (0.0%) |
| Present and fully covered | 0 (0.0%) | 0 (0.0%) | 0 (0.0%) |  | 1 (50.0%) | 0 (0.0%) | | 2 (22.2%) | 0 (0.0%) | 0 (0.0%) | 0 (0.0%) |
| **Non-project cabinet – Store Packaged Bought Foods** |  |  |  |  |  |  | |  |  |  |  |
| Present, but no cover | 2 (100.0%) |  |  |  | 0 (0.0%) | 6 (66.7%) | | 7 (58.3%) | 7 (63.6%) | 1 (20.0%) | 1 (14.3%) |
| Present, but partially covered | 0 (0.0%) |  |  |  | 0 (0.0%) | 0 (0.0%) | | 0 (0.0%) | 0 (0.0%) | 0 (0.0%) | 0 (0.0%) |
| Present and fully covered | 0 (0.0%) |  |  |  | 1 (100.0%) | 3 (33.3%) | | 5 (41.7%) | 4 (36.4%) | 4 (80.0%) | 6 (85.7%) |
| **Under bed – Cooked Food** |  |  |  |  |  |  | |  |  |  |  |
| Present, but no cover | 1 (50.0%) | 2 (66.7%) | 1 (50.0%) |  |  | 3 (18.8%) | | 2 (18.2%) | 1 (11.1%) | 0 (0.0%) | 0 (0.0%) |
| Present, but partially covered | 1 (50.0%) | 1 (33.3%) | 0 (0.0%) |  |  | 3 (18.8%) | | 1 (9.1%) | 1 (11.1%) | 1 (16.7%) | 0 (0.0%) |
| Present and fully covered | 0 (0.0%) | 0 (0.0%) | 1 (50.0%) |  |  | 10 (62.5%) | | 8 (72.7%) | 7 (77.8%) | 5 (83.3%) | 7 (100.0%) |
| **Under bed – Ingredients** |  |  |  |  |  |  | |  |  |  |  |
| Present, but no cover | 18 (78.3%) | 14 (51.9%) | 18 (58.1%) | 16 (39.0%) | 15 (39.5%) | 23 (69.7%) | | 22 (59.5%) | 27 (57.4%) | 23 (48.9%) | 15 (30.0%) |
| Present, but partially covered | 1 (4.3%) | 2 (7.4%) | 2 (6.5%) | 4 (9.8%) | 4 (10.5%) | 3 (9.1%) | | 2 (5.4%) | 0 (0.0%) | 3 (6.4%) | 12 (24.0%) |
| Present and fully covered | 4 (17.4%) | 11 (40.7%) | 11 (35.5%) | 21 (51.2%) | 19 (50.0%) | 7 (21.2%) | | 13 (35.1%) | 20 (42.6%) | 21 (44.7%) | 23 (46.0%) |
| **Under bed – Raw Fruits/Vegetables** |  |  |  |  |  |  | |  |  |  |  |
| Present, but no cover | 27 (90.0%) | 17 (60.7%) | 18 (60.0%) | 22 (61.1%) | 17 (47.2%) | 30 (73.2%) | | 31 (79.5%) | 35 (72.9%) | 25 (62.5%) | 28 (56.0%) |
| Present, but partially covered | 3 (10.0%) | 7 (25.0%) | 8 (26.7%) | 11 (30.6%) | 15 (41.7%) | 10 (24.4%) | | 6 (15.4%) | 5 (10.4%) | 11 (27.5%) | 18 (36.0%) |
| Present and fully covered | 0 (0.0%) | 4 (14.3%) | 4 (13.3%) | 3 (8.3%) | 4 (11.1%) | 1 (2.4%) | | 2 (5.1%) | 8 (16.7%) | 4 (10.0%) | 4 (8.0%) |
| **Under bed – Store Packaged Bought Foods** |  |  |  |  |  |  | |  |  |  |  |
| Present, but no cover | 0 (0.0%) | 1 (33.3%) | 0 (0.0%) |  |  | 5 (71.4%) | | 1 (12.5%) | 1 (11.1%) | 1 (20.0%) | 0 (0.0%) |
| Present, but partially covered | 0 (0.0%) | 0 (0.0%) | 1 (33.3%) |  |  | 0 (0.0%) | | 0 (0.0%) | 0 (0.0%) | 0 (0.0%) | 0 (0.0%) |
| Present and fully covered | 1 (100.0%) | 2 (66.7%) | 2 (66.7%) |  |  | 2 (28.6%) | | 7 (87.5%) | 8 (88.9%) | 4 (80.0%) | 3 (100.0%) |
| **Open shelf – Cooked Food** |  |  |  |  |  |  | |  |  |  |  |
| Present, but no cover | 5 (45.5%) | 3 (42.9%) |  | 0 (0.0%) | 0 (0.0%) | 5 (5.9%) | | 5 (6.8%) | 3 (3.6%) | 1 (1.3%) | 1 (1.2%) |
| Present, but partially covered | 3 (27.3%) | 2 (28.6%) |  | 1 (100.0%) | 1 (100.0%) | 40 (47.1%) | | 28 (37.8%) | 28 (33.3%) | 20 (26.3%) | 29 (34.5%) |
| Present and fully covered | 3 (27.3%) | 2 (28.6%) |  | 0 (0.0%) | 0 (0.0%) | 40 (47.1%) | | 41 (55.4%) | 53 (63.1%) | 55 (72.4%) | 54 (64.3%) |
| **Open shelf – Ingredients** |  |  |  |  |  |  | |  |  |  |  |
| Present, but no cover | 13 (72.2%) | 11 (64.7%) | 7 (41.2%) | 4 (22.2%) | 8 (47.1%) | 23 (65.7%) | | 16 (41.0%) | 16 (36.4%) | 13 (21.0%) | 13 (23.2%) |
| Present, but partially covered | 1 (5.6%) | 0 (0.0%) | 3 (17.6%) | 2 (11.1%) | 3 (17.6%) | 4 (11.4%) | | 2 (5.1%) | 1 (2.3%) | 2 (3.2%) | 3 (5.4%) |
| Present and fully covered | 4 (22.2%) | 6 (35.3%) | 7 (41.2%) | 12 (66.7%) | 6 (35.3%) | 8 (22.9%) | | 21 (53.8%) | 27 (61.4%) | 47 (75.8%) | 40 (71.4%) |
| **Open shelf – Raw Fruits/Vegetables** |  |  |  |  |  |  | |  |  |  |  |
| Present, but no cover | 16 (80.0%) | 9 (56.3%) | 8 (66.7%) | 5 (38.5%) | 7 (50.0%) | 23 (74.2%) | | 8 (40.0%) | 14 (56.0%) | 16 (59.3%) | 13 (86.7%) |
| Present, but partially covered | 4 (20.0%) | 4 (25.0%) | 3 (25.0%) | 7 (53.8%) | 6 (42.9%) | 4 (12.9%) | | 2 (10.0%) | 5 (20.0%) | 6 (22.2%) | 1 (6.7%) |
| Present and fully covered | 0 (0.0%) | 3 (18.8%) | 1 (8.3%) | 1 (7.7%) | 1 (7.1%) | 4 (12.9%) | | 10 (50.0%) | 6 (24.0%) | 5 (18.5%) | 1 (6.7%) |
| **Open shelf – Store Packaged Bought Foods** |  |  |  |  |  |  | |  |  |  |  |
| Present, but no cover | 3 (42.9%) | 0 (0.0%) | 1 (33.3%) | 1 (33.3%) | 0 (0.0%) | 11 (34.4%) | | 5 (15.6%) | 7 (16.7%) | 6 (15.8%) | 8 (25.0%) |
| Present, but partially covered | 0 (0.0%) | 0 (0.0%) | 0 (0.0%) | 0 (0.0%) | 1 (100.0%) | 5 (15.6%) | | 3 (9.4%) | 0 (0.0%) | 1 (2.6%) | 0 (0.0%) |
| Present and fully covered | 4 (57.1%) | 3 (100.0%) | 2 (66.7%) | 2 (66.7%) | 0 (0.0%) | 16 (50.0%) | | 24 (75.0%) | 35 (83.3%) | 31 (81.6%) | 24 (75.0%) |
