# Supplementary material for: Evaluating the impact of a low-cost food storage intervention on complementary food contamination and diarrheal disease in low-income urban households: A randomized controlled trial in Dhaka, Bangladesh
Source: PLOS Glob Public Health. 2026 Jun 11;6(6):e0005883. doi: 10.1371/journal.pgph.0005883 (PMC13257995; doi:10.1371/journal.pgph.0005883)
Supplement: Attachment — Submitted filename: Response_to_Reviewers_PGPH-D-26-00103.docx [file pgph.0005883.s008.docx]

**PLOS Global Public Health — Response to Reviewers**

Manuscript ID: PGPH-D-26-00103

Date: April 8, 2026

We thank the Academic Editor and both Reviewers for their constructive comments. We have addressed each point in full below. All changes are reflected in the Revised Manuscript with Track Changes and the clean Manuscript submitted alongside this letter.

**Journal Requirements**

**Requirement 1 — Trial Registration**

*If you are submitting a clinical trial, it must be registered in a recognized international registry. Please record the registration number in your submission Abstract, and include both the registration number and direct URL in the Methods section. Please also provide the complete date range for participant recruitment and follow-up in the Methods section.*

**Response:** This trial was retrospectively registered with ClinicalTrials.gov (NCT07332078; https://clinicaltrials.gov/study/NCT07332078). The trial was registered on January 12, 2026, after completion of data collection, due to administrative delays in registry processing. We have made the following four additions to the revised manuscript:

(1) The following trial registration line has been added to the end of the Abstract, before the Keywords: “Trial registration: ClinicalTrials.gov, NCT07332078. Registered 12 January 2026 (retrospectively registered); https://clinicaltrials.gov/study/NCT07332078.”

(2) The complete date range for participant recruitment and follow-up is confirmed in the Data Collection subsection of the Methods, which already stated: “Baseline data collection began on August 11, 2024” and “The final data collection visit (PI-5) was conducted on November 3, 2024.” These dates were present in the original submission and required no new text.

(3) The following sentence has been added to the Trial Registration subsection of the Methods to make the registration timeline explicit: “The trial was registered on January 12, 2026, after completion of data collection.”

(4) The following confirmation statement has been added to the Trial Registration subsection: “The authors confirm that all ongoing and related trials for this intervention are registered.”

**Manuscript change:** Trial registration line (NCT07332078, date, URL) added to end of Abstract. Registration date sentence added to Trial Registration subsection. Date range already present in Data Collection subsection (August 11 – November 3, 2024). Confirmation statement added to Trial Registration subsection.

**Requirement 2a — Figure 2: Identifiable Person**

*Figure 2 includes an image of an identifiable person. Please provide written confirmation or release forms, signed by the subject(s) (or their guardian), giving permission to be photographed and to have their images published under a Creative Commons license. Otherwise, we kindly request that you remove the photograph.*

**Response:** Figure 2 comprises the two illustrated behavior-change communication (BCC) handouts distributed to intervention households. The updated figure file submitted with this revision has been deidentified: all recognizable faces, including the child visible in the top-left panel and the caregiver visible in the right-hand panel, have been blurred. No individual is identifiable in the revised figure. A photo release form is therefore not required. The revised Figure 2 file is included in the figure resubmission.

**Manuscript change:** Figure 2 resubmitted with all faces blurred. No manuscript text change required.

**Requirement 2b — Figure 2: Photographer Confirmation**

*Figure2.tiff: Please confirm (a) that you are the photographer; or (b) provide written permission from the photographer to publish the photo(s) under our CC-BY 4.0 license.*

**Response:** All photographs included in Figure 2 were taken by members of the icddr,b study team during data collection. The corresponding author (MJR) and icddr,b co-investigators hold full rights to these images. We confirm that we are the photographers and grant permission to publish under the CC-BY 4.0 license. No third-party copyright clearance is required.

**Manuscript change:** No manuscript change required. Photographer confirmation provided here and in the submission cover letter.

**Requirement 3 — Supporting Information Legends**

*We have noticed that you have uploaded Supporting Information files, but you have not included a list of legends. Please add a full list of legends for your Supporting Information files after the references list.*

**Response:** A “Supporting Information” section has been added immediately after reference 55 in the revised manuscript. The legends appear exactly as follows:

*Supplementary Figure 1:* Meatsafe of the type distributed to intervention households.

*Supplementary Figure 2:* Examples of pre-existing non-project cabinets for food storage.

*Supplementary Figure 3:* Shelves that are open, and not protected in any way other than being elevated surfaces.

*Supplementary Table 1: E. coli* contamination levels by study arm and visit.

*Supplementary Table 2:* 7-Day diarrhea prevalence by study arm and visit.

*Supplementary Table 3:* Type of food and storage locations across the intervention and control arms at each post-intervention visits.

*Supplementary Table 4:* Type of food and cover status in different locations across intervention and control at each post-intervention visit.

**Manuscript change:** Supporting Information section with full legends list added after reference 55 (two new references added in response to Reviewer 1; see below).

**Reviewer 1**

**Comment 1.1 — Reconciling Analysis Method with Pre-specified Protocol**

*They conducted intention-to-treat analyses. They estimated prevalence ratio (PR) of high E. coli contamination and diarrhea using a population-averaged, modified Poisson regression with robust standard errors clustered at the household level. This appeared to be different than the intended analysis noted in the supplement protocol PR_24022. This reviewer had a challenge reconciling the sample size (households) and analysis intents of this protocol with the written manuscript. Some clarification should be attempted.*

**Response:** We thank Reviewer 1 for this careful observation. The pre-specified analysis in protocol PR-24022 called for intention-to-treat estimation of prevalence ratios of the binary contamination outcome, accounting for clustering at the household level (pre-specified intracluster correlation coefficient (ICC) = 0.20 in the sample size calculation). The manuscript implements this as a population-averaged modified Poisson regression with robust standard errors clustered at the household level. This estimator was selected because: (1) it directly produces the prevalence ratio on the relative scale for binary outcomes; (2) it avoids the convergence problems that frequently arise with log-binomial models in trials with moderate effect sizes; and (3) the sandwich variance estimator correctly handles within-household correlation across repeated food samples without requiring parametric assumptions about the random-effects distribution. This approach is consistent with the analytic intent of the protocol and with current methodological guidance for clustered RCTs with binary outcomes [54, 55]. We have revised the Data Analysis section of the Methods to make this reasoning explicit, and have added the two supporting references (references 54 and 55) to the reference list.

The two new references added are:

54. Zou G: **A modified Poisson regression approach to prospective studies with binary data.** *Am J Epidemiol* 2004, **159**(7):702–706.

55. Yelland LN, Salter AB, Ryan P: **Performance of the modified Poisson regression approach for estimating relative risks from clustered prospective data.** *Am J Epidemiol* 2011, **174**(8):984–992.

**Manuscript change:** Data Analysis section revised to open with an explicit statement that the modified Poisson regression implements the pre-specified ITT analysis, followed by a three-part justification for the estimator choice over log-binomial regression. Citation [54, 55] added at the end of the justification passage. Both references added to the reference list as references 54 and 55. The Supporting Information legends section, previously numbered after reference 53, now follows reference 55.

**Reviewer 2**

**Comment 2.1 — Randomization: Insufficient Description of Mechanism**

*Households were assigned randomly, but there is not enough explanation of exactly how that was done.*

**Response:** We thank the reviewer for this comment. The original manuscript stated that randomization occurred but did not describe the mechanism. We have revised the Randomization section to meet CONSORT reporting standards. The section now reads: “After collecting baseline data, a central coordinator randomly generated the allocation sequence using a computer-based random number generator in Stata, with no blocking or stratification. Household IDs were entered sequentially in the order of enrollment, and each was assigned to intervention or control with equal probability. To reduce the risk of contamination between arms, enrolled households were required to be at least 100 meters apart; enumerators moved in a clockwise direction from a fixed starting point in the settlement, knocking on every alternate household, to maintain geographic spacing during recruitment. Allocation was implemented centrally by a coordinator who was not involved in data collection; field enumerators learned a household’s assignment only at the point of intervention delivery, one to two days after the baseline visit.”

**Manuscript change:** Randomization section substantially revised to specify: (1) sequence generation — computer-based random number generator in Stata, no blocking or stratification, equal probability of assignment; (2) geographic spillover prevention — 100m minimum spacing, clockwise systematic recruitment from a fixed starting point; (3) allocation concealment — central coordinator separate from data collection team; (4) timing of disclosure — field enumerators informed only at point of intervention delivery, one to two days post-baseline.

**Comment 2.2 — Conclusion: Technology Recommendations Not Evaluated in Study**

*The final paragraph of the conclusion introduces recommendations about technologies that were not evaluated in the study, and the connection to the trial findings is speculative. It could be framed as future research directions rather than direct implications of the results.*

**Response:** We agree with the reviewer. The original final sentence drew direct programmatic implications from technologies not evaluated in this trial. We have revised it to frame these technologies as candidates for future investigation. The revised Conclusion now closes with: “Future research could explore whether pairing more intensive and sustained BCC with affordable thermal technologies such as solar refrigerators, direct-drive fridges, phase-change–salt cookers, and hybrid solar–LPG stoves [50–53] yields greater reductions in food contamination and diarrhea in similar settings, and whether such combinations are feasible and scalable in low-income urban contexts.” The first two sentences of the Conclusion, which speak directly to the trial’s findings, are unchanged.

**Manuscript change:** Final sentence of the Conclusion revised from a direct programmatic recommendation to a future research framing. Original: “Future efforts should pair intensive BCC strategy with more advanced, but affordable, technologies...that enable safe storage and reheating even in off-grid settings.” Revised: “Future research could explore whether pairing more intensive and sustained BCC with affordable thermal technologies...yields greater reductions in food contamination and diarrhea in similar settings, and whether such combinations are feasible and scalable in low-income urban contexts.”

**Comment 2.3 — Data Availability**

*Reviewer 2 indicated that data underlying the findings are not fully available (answered “No” to the data availability question).*

**Response:** The deidentified dataset is available from the corresponding author upon reasonable request, as stated in the Data Availability Statement. Public deposition was not undertaken due to ethical and privacy considerations specific to human subjects research conducted in informal urban settlements in Dhaka. Both the UC Berkeley Committee for Protection of Human Subjects (Protocol #2024-04-17365) and the icddr,b Ethical Review Committee (PR-24022) approved the data management plan on the basis that participant-level data would not be publicly deposited, given the vulnerability of the study population and the sensitivity of household-level data collected in an informal settlement context. The Data Availability Statement in the manuscript explicitly states the restriction reason and provides the corresponding author’s contact information for data access requests. This arrangement is consistent with PLOS’s data policy provisions for data with justified access restrictions.

**Manuscript change:** No manuscript change required. The Data Availability Statement already specifies the ethical restriction and the request mechanism (corresponding author contact).

We believe these revisions fully address all points raised by the Academic Editor and both Reviewers. We are grateful for the careful and constructive review and hope the revised manuscript is now suitable for acceptance.

Sincerely,

Musarrat Jabeen Rahman, PhD, MPH

Corresponding Author

rahman.musarrat@gmail.com
