# Supplementary material for: Evaluating the impact of a low-cost food storage intervention on complementary food contamination and diarrheal disease in low-income urban households: A randomized controlled trial in Dhaka, Bangladesh
Source: PLOS Glob Public Health. 2026 Jun 11;6(6):e0005883. doi: 10.1371/journal.pgph.0005883 (PMC13257995; doi:10.1371/journal.pgph.0005883)
Supplement: Peer review history

# Peer review: Decision Letter 0

**DOI:** 10.1371/journal.pgph.0005883.r001

**Authors:** Rebeca Sultana

12 Mar 2026

PGPH-D-26-00103

Evaluating the Impact of a Low-Cost Food Storage Intervention on Complementary Food Contamination and Diarrheal Disease in Low-Income Urban Households: A Randomized Controlled Trial in Dhaka, Bangladesh

PLOS Global Public Health

Dear Authors,

Thank you for submitting your manuscript to PLOS Global Public Health. After careful consideration, we feel that it has merit but does not fully meet PLOS Global Public Health’s publication criteria as it currently stands. Therefore, we invite you to submit a revised version of the manuscript that addresses the points raised during the review process.

Please submit your revised manuscript by 28 March, 2026. If you will need more time than this to complete your revisions, please reply to this message or contact the journal office at globalpubhealth@plos.org. When you're ready to submit your revision, log on to https://www.editorialmanager.com/pgph/ and select the 'Submissions Needing Revision' folder to locate your manuscript file.

Please include the following items when submitting your revised manuscript:

- A letter that responds to each point raised by the editor and reviewer(s). You should upload this letter as a separate file labeled 'Response to Reviewers'.
- A marked-up copy of your manuscript that highlights changes made to the original version. You should upload this as a separate file labeled 'Revised Manuscript with Track Changes'.
- An unmarked version of your revised paper without tracked changes. You should upload this as a separate file labeled 'Manuscript'.

Guidelines for resubmitting your figure files are available below the reviewer comments at the end of this letter.

We look forward to receiving your revised manuscript.

Kind regards,

Academic Editor

PLOS Global Public Health

Journal Requirements:

1. If you are submitting a clinical trial, it must be registered in a recognized international registry, e.g. ClinicalTrials.gov or the International Standard Randomized Controlled Trial Number (ISRCTN) Registry. Please record the registration number in your submission Abstract, and include both the registration number and direct URL to the trial registration in the Methods section of your manuscript. Please also provide the complete date range for participant recruitment and follow-up in the Methods section of your manuscript.

If you have not yet registered your trial in an appropriate registry, we now require you to do so and will need confirmation of the trial registry number before we can pass your paper to the next stage of review. Please include in the Methods section of your paper your reasons for not registering this study before enrolment of participants started. Please confirm that all related trials are registered by stating: “The authors confirm that all ongoing and related trials for this drug/intervention are registered”.

2. Some material included in your submission may be copyrighted. According to PLOS’s copyright policy, authors who use figures or other material (e.g., graphics, clipart, maps) from another author or copyright holder must demonstrate or obtain permission to publish this material under the Creative Commons Attribution 4.0 International (CC BY 4.0) License used by PLOS journals. Please closely review the details of PLOS’s copyright requirements here: PLOS Licenses and Copyright. If you need to request permissions from a copyright holder, you may use PLOS's Copyright Content Permission form.

Please respond directly to this email or email the journal office and provide any known details concerning your material's license terms and permissions required for reuse, even if you have not yet obtained copyright permissions or are unsure of your material's copyright compatibility.

Potential Copyright Issues:

a. Figure 2 includes an image of an identifiable person. Please provide written confirmation or release forms, signed by the subject(s) (or their guardian), giving permission to be photographed and to have their images published under a Creative Commons license. You may upload permission forms to your submission file inventory as item type 'Other'. Otherwise, we kindly request that you remove the photograph.

b. Figure2.tiff: Please confirm (a) that you are the photographer; or (b) provide written permission from the photographer to publish the photo(s) under our CC-BY 4.0 license.

3. We have noticed that you have uploaded Supporting Information files, but you have not included a list of legends. Please add a full list of legends for your Supporting Information files after the references list.

If the reviewer comments include a recommendation to cite specific previously published works, please review and evaluate these publications to determine whether they are relevant and should be cited. There is no requirement to cite these works unless the editor has indicated otherwise.

Additional Editor Comments (if provided):

[Note: HTML markup is below. Please do not edit.]

Reviewers' comments:

Reviewer's Responses to Questions

**Comments to the Author**

1. Does this manuscript meet PLOS Global Public Health’s publication criteria? Is the manuscript technically sound, and do the data support the conclusions? The manuscript must describe methodologically and ethically rigorous research with conclusions that are appropriately drawn based on the data presented.

Reviewer #1: Partly

Reviewer #2: Partly

**********

2. Has the statistical analysis been performed appropriately and rigorously?

Reviewer #1: Yes

Reviewer #2: I don't know

**********

3. Have the authors made all data underlying the findings in their manuscript fully available (please refer to the Data Availability Statement at the start of the manuscript PDF file)?

The PLOS Data policy requires authors to make all data underlying the findings described in their manuscript fully available without restriction, with rare exception. The data should be provided as part of the manuscript or its supporting information, or deposited to a public repository. For example, in addition to summary statistics, the data points behind means, medians and variance measures should be available. If there are restrictions on publicly sharing data—e.g. participant privacy or use of data from a third party—those must be specified.

Reviewer #1: Yes

Reviewer #2: No

**********

4. Is the manuscript presented in an intelligible fashion and written in standard English?

PLOS Global Public Health does not copyedit accepted manuscripts, so the language in submitted articles must be clear, correct, and unambiguous. Any typographical or grammatical errors should be corrected at revision, so please note any specific errors here.

Reviewer #1: Yes

Reviewer #2: Yes

**********

5. Review Comments to the Author

Please use the space provided to explain your answers to the questions above. You may also include additional comments for the author, including concerns about dual publication, research ethics, or publication ethics. (Please upload your review as an attachment if it exceeds 20,000 characters)

Reviewer #1: There were reasonably designed with sample size and power considerations. However, some of the design and analysis strategies in the included supplemental protocol and the actual manuscript have to be reconciled as noted below.

Assuming a conservative 50% compliance rate with intervention, they estimated a 25% reduction in contamination prevalence in the intervention arm, from 30.4% t 150 o 22.8%. To detect this 151 effect with 80% power and a two-sided α of 0.05, over five repeated food-sampling visits 152 per household and an intra cluster correlation coefficient (ICC) of 0.04 , 153 assuming 15% attrition they estimate a sample size was 145 households per study

They conducted intention-to-treat analyses. They estimated prevalence ratio (PR) of high E. coli contamination and diarrhea using a population-averaged, modified Poisson regression with robust standard errors clustered at the household level. This appeared to be different than the intended analysis noted in the supplement protocol PR_24022. However, the results were seen from the text and Figures 3 to 5. This reviewer had a challenge reconciling the sample size (households) and analysis intents of this protocol with the written manuscript. The overall conclusion on page 24 of the manuscript appeared to follow from the analysis reported. Some clarification should be attempted.

Reviewer #2: The manuscript is generally good, readable and well structured, but it would benefit from careful framing/positioning and language editing before it can be accepted. More concretely, there are some issues that need to be addressed before acceptance. Eg: Households were assigned randomly, but there is not enough explanation of exactly how that was done. The final paragraph of the conclusion introduces recommendations about technologies that were not evaluated in the study, and the connection to the trial findings is speculative. It could be framed as future research directions rather than direct implications of the results

**********

6. PLOS authors have the option to publish the peer review history of their article (what does this mean?). If published, this will include your full peer review and any attached files.

**Do you want your identity to be public for this peer review?**  If you choose “no”, your identity will remain anonymous but your review may still be made public.

For information about this choice, including consent withdrawal, please see our Privacy Policy.

Reviewer #1: No

Reviewer #2: **Yes:**  Winifred.Addo-Cobbiah

**********

[NOTE: If reviewer comments were submitted as an attachment file, they will be attached to this email and accessible via the submission site. Please log into your account, locate the manuscript record, and check for the action link "View Attachments". If this link does not appear, there are no attachment files.]

-->Figure Resubmissions:

While revising your submission, we strongly recommend that you use PLOS’s NAAS tool (https://ngplosjournals.pagemajik.ai/artanalysis) to test your figure files. NAAS can convert your figure files to the TIFF file type and meet basic requirements (such as print size, resolution), or provide you with a report on issues that do not meet our requirements and that NAAS cannot fix.

After uploading your figures to PLOS’s NAAS tool - https://ngplosjournals.pagemajik.ai/artanalysis, NAAS will process the files provided and display the results in the "Uploaded Files" section of the page as the processing is complete. If the uploaded figures meet our requirements (or NAAS is able to fix the files to meet our requirements), the figure will be marked as "fixed" above. If NAAS is unable to fix the files, a red "failed" label will appear above. When NAAS has confirmed that the figure files meet our requirements, please download the file via the download option, and include these NAAS processed figure files when submitting your revised manuscript.

# Author comment: Author response to Decision Letter 1

**DOI:** 10.1371/journal.pgph.0005883.r002

19 Apr 2026

Attachment Submitted filename: Response_to_Reviewers_PGPH-D-26-00103.docx

# Editor report: Decision Letter 1

**DOI:** 10.1371/journal.pgph.0005883.r003

**Authors:** Rebeca Sultana

6 May 2026

Evaluating the Impact of a Low-Cost Food Storage Intervention on Complementary Food Contamination and Diarrheal Disease in Low-Income Urban Households: A Randomized Controlled Trial in Dhaka, Bangladesh

PGPH-D-26-00103R1

Dear Authors,

We are pleased to inform you that your manuscript 'Evaluating the Impact of a Low-Cost Food Storage Intervention on Complementary Food Contamination and Diarrheal Disease in Low-Income Urban Households: A Randomized Controlled Trial in Dhaka, Bangladesh' has been provisionally accepted for publication in PLOS Global Public Health.

Before your manuscript can be formally accepted you will need to complete some formatting changes, which you will receive in a follow up email. A member of our team will be in touch with a set of requests.

Please note that your manuscript will not be scheduled for publication until you have made the required changes, so a swift response is appreciated.

IMPORTANT: The editorial review process is now complete. PLOS will only permit corrections to spelling, formatting or significant scientific errors from this point onwards. Requests for major changes, or any which affect the scientific understanding of your work, will cause delays to the publication date of your manuscript.

If your institution or institutions have a press office, please notify them about your upcoming paper to help maximize its impact. If they'll be preparing press materials, please inform our press team as soon as possible -- no later than 48 hours after receiving the formal acceptance. Your manuscript will remain under strict press embargo until 2 pm Eastern Time on the date of publication. For more information, please contact globalpubhealth@plos.org.

Thank you again for supporting Open Access publishing; we are looking forward to publishing your work in PLOS Global Public Health.

Best regards,

Rebeca Sultana

Academic Editor

PLOS Global Public Health
